# Superhard Boron-Rich Boron Carbide with Controlled Degree of Crystallinity

**DOI:** 10.3390/ma13163622

**Published:** 2020-08-16

**Authors:** Kallol Chakrabarty, Wei-Chih Chen, Paul A. Baker, Vineeth M. Vijayan, Cheng-Chien Chen, Shane A. Catledge

**Affiliations:** Department of Physics, University of Alabama at Birmingham, Birmingham, AL 35294, USA; kallol89@uab.edu (K.C.); weichih@uab.edu (W.-C.C.); pabaker@uab.edu (P.A.B.); vineeth@uab.edu (V.M.V.); chencc@uab.edu (C.-C.C.)

**Keywords:** ceramics/coating materials, chemical synthesis, vapor deposition, mechanical properties, crystal structure, computer simulation

## Abstract

Superhard boron-rich boron carbide coatings were deposited on silicon substrates by microwave plasma chemical vapor deposition (MPCVD) under controlled conditions, which led to either a disordered or crystalline structure, as measured by X-ray diffraction. The control of either disordered or crystalline structures was achieved solely by the choice of the sample being placed either directly on top of the sample holder or within an inset of the sample holder, respectively. The carbon content in the B-C bonded disordered and crystalline coatings was 6.1 at.% and 4.5 at.%, respectively, as measured by X-ray photoelectron spectroscopy. X-ray diffraction analysis of the crystalline coating provided a good match with a B_50_C_2_-type structure in which two carbon atoms replaced boron in the α-tetragonal B_52_ structure, or in which the carbon atoms occupied different interstitial sites. Density functional theory predictions were used to evaluate the dynamical stability of the potential B_50_C_2_ structural forms and were consistent with the measurements. The measured nanoindentation hardness of the coatings was as high as 64 GPa, well above the 40 GPa threshold for superhardness.

## 1. Introduction

Materials based on the light elements of carbon, nitrogen, oxygen, and boron with strong covalent bonds comprise some of the hardest known materials [1,2,3]. These light elements can form short bond lengths with each other and are inclined to form directional covalent bonds, making the structures they form difficult to compress or distort. Boron carbide is the third hardest material after diamond and cubic boron nitride. Elemental boron can form base structures consisting of B_12_ icosahedra, B_6_ octahedra, linear atomic chains, and/or atomic clusters in a three-dimensional network [4,5,6,7]. There are multiple possible arrangements of icosahedra, together with additional structural elements, that can form during material processing routes [3,8,9]. The common stoichiometry of boron carbide includes B_13_C_2_, B_12_C_3_, and B_4_C, as well as some structures that are similar to B_12_C_3_ [3,8]. Some notable boron carbide crystal structures are tetragonal, including B_50_C_2_, B_50_C, B_48_C_3_, B_51_C, and B_49_C_3_ [9,10]. Several theoretical and experimental studies have suggested that, as the boron to carbon ratio varies, the atomic bonding, electron density, mechanical properties, and lattice constants of boron carbide change significantly [11,12,13,14,15]. Thermodynamically, the most stable forms of boron carbide are the α-tetragonal and rhombohedral crystal structures [11,16]. Boron-rich carbides are most known for their high hardness, extreme abrasion resistance, high melting point, thermal stability, high mechanical strength, high neutron absorption, and their ability to function in extreme conditions of pressure, temperature, and corrosive environments [11,16,17,18,19,20,21]. These properties make boron carbide suitable in refractory applications, medical applications, fast breeders, lightweight armors, ballistic armors, as cutting tools, as an abrasive powder, and for high-temperature thermoelectric conversion. Depending on the growth process, a wide range of boron carbide stoichiometries can be created with desirable chemical and electrical properties, opening the door to other potential applications.

Several techniques and experimental conditions have been used to synthesize boron carbides using a variety of boron precursors [22,23]. The preparation of novel boron-rich boron carbide was reported from high-pressure high-temperature (HPTP) cells which yield very small volumes of material [22]. These methods are not scalable for producing coatings for large areas and it can be difficult to control impurities. Instead, chemical vapor deposition (CVD) has proven to be a scalable technology for synthesizing a wide range of coating materials including boron carbide with large area uniformity [23,24]. A primary challenge of CVD is to find the optimum conditions that are favorable for the growth of the desired phase.

The objective of this work is to investigate deposition conditions leading to superhard boron-rich boron carbide coatings, to evaluate their structure experimentally and by density functional theory, and to demonstrate control of boron carbide crystallinity by the appropriate choice of sample height in the plasma. To this end, superhard boron-rich boron carbide coatings were synthesized by microwave plasma chemical vapor deposition (MPCVD), using conditions of chamber pressure and microwave power that we found led to copious amounts of atomic boron plasma emission [9,25]. Reactant gases for the growth of the boron carbide coatings include H_2_ and B_2_H_6_, but little is known of the plasma species and underlying the spectroscopic aspects responsible for boron carbide growth in its various structural forms. Investigation of the excited state plasma species from optical emission spectroscopy in our MPCVD system shows that emission from atomic boron is highest at low chamber pressure and high microwave power [25,26]. Copious amounts of atomic boron in the plasma may be beneficial under some growth conditions for producing high hardness boron-rich carbides, such as B_50_C_2_, B_50_C, B_48_C_3_, B_51_C, B_49_C_3_, or many other forms of boron-rich carbide. The enhanced atomic boron emission in the plasma with the MPCVD conditions used in the current study yielded a higher boron content in the boron carbide coatings with measured superhardness (i.e., hardness greater than 40 GPa). In addition, we report that with very modest changes in the sample stage design (and thus the proximity of the sample to the plasma), the degree of crystallinity in the coating could vary significantly.

## 2. Materials and Methods

### 2.1. MPCVD Process

**Figure 1:**
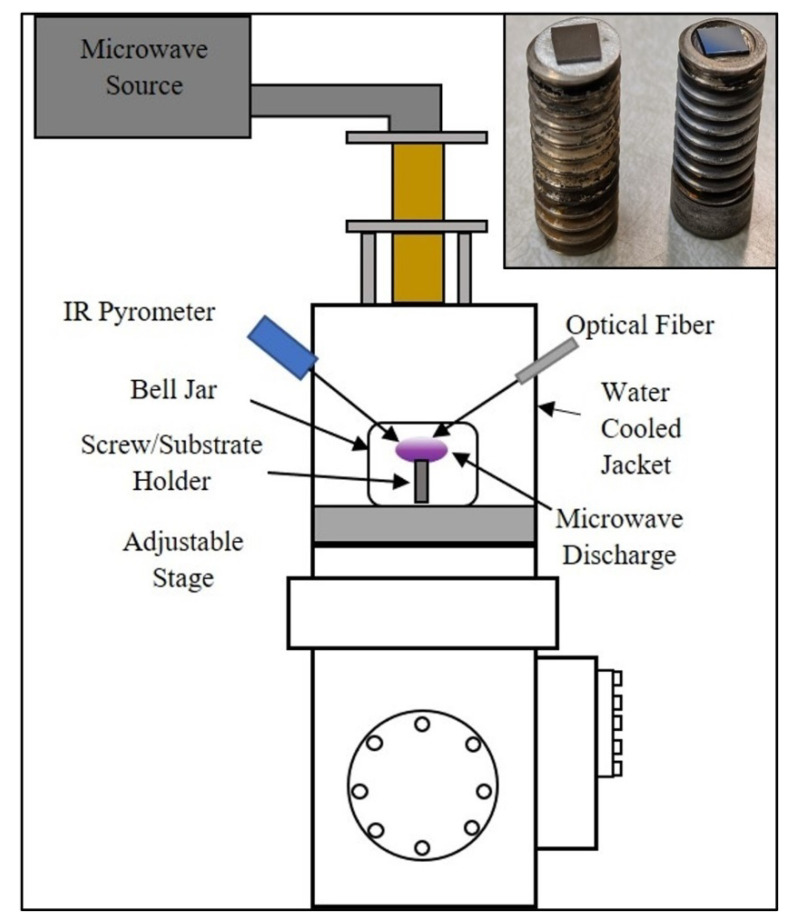
Schematic of the 6 kW Microwave Plasma Chemical Vapor Deposition (MPCVD) chamber showing plasma confined within a quartz bell jar. The inset shows how the substrates were placed on the substrate holder for sample BC-1 (flat screw) and BC-2 (screw with inset).

Boron carbide coatings were grown in a microwave plasma chemical vapor deposition (MPCVD) system, as shown in Figure 1 (Wavemat Inc. Plymouth, MI, USA). The sample surface was heated by direct contact with the plasma. Both the sample stage and outer resonance cavity jacket were water cooled. A quartz bell jar isolates the low-pressure plasma environment from the resonance cavity. N-type (100)-oriented silicon substrates with 525 μm thickness were placed on the surface of a 0.5” diameter molybdenum screw placed along the central axis of the bell jar. Sample substrates were cleaned in acetone, methanol, and distilled water. The microwave power was 1 kW and the chamber pressure was 15 Torr. Hydrogen (H_2_) was used as the carrier gas and a diborane mixture (90% H_2_, 10% B_2_H_6_, and ppm carbon) as the reactive gas. Low levels of residual carbon have been found to appear consistently in high-boron deposited films. The gas flow rates were: 500 standard cubic centimeters per minute (SCCM) of hydrogen and 1 SCCM of the diborane mixture. Two types of samples were grown at an average substrate temperature of 825 °C with the same experimental conditions, with the exception of how the silicon substrate was positioned in/on a flat molybdenum screw, as shown in Figure 1. For samples designated as BC-1, the silicon substrate rested on the flat surface of the molybdenum screw, whereas for samples designated as BC-2, the silicon substrate rested within a 0.5 mm inset of the screw face such that the substrate was flush with the surrounding Mo surface. BC-1 and BC-2 samples were grown at the same average temperature of 825 ± 25 °C.

### 2.2. Characterization Techniques

Samples were characterized using X-ray photoelectron spectroscopy (XPS), glancing angle X-ray diffraction (XRD), Raman spectroscopy, Fourier transform infrared spectroscopy (FTIR), nanoindentation, and scanning electron microscopy (SEM). The XPS instrumentation was a Phi Electronics Versaprobe 5000 (Phi Electronics, Chanhassen, MN, USA), equipped with a micro-focused Al monochromatic source (λ = 1486.6 eV) and a dual anode conventional X-ray source with a neutralizer. Survey spectra were taken with an incident energy of 1253.6 eV and both sources were used for data collection. The XRD pattern was obtained by a Panalytical Empyrean X-ray diffractometer (Copper K_α1_, λ = 1.54059 Å) (Malvern, Panalytical, Almelo, Nederland). XRD patterns were acquired using a glancing angle 2-theta scan with an angle of incidence of 1 degree. The diffraction optics included a hybrid monochromator with a 1/8° divergence slit and a 1/16° anti-scattering slit and a parallel plate collimator on the diffracted beam path with a proportional detector. HighScore Plus software (version 4.8) was used to analyze phase structure. Rietveld refinement was used to find the lattice constants of the BC-2 sample. Raman spectra were collected with a micro-Raman spectrometer (Dilor XY, Lille, France) with a 532 nm laser, 1200 groove/mm grating, and a 100× microscope objective. The Bruker alpha FTIR spectrometer (Bruker Corporation, Billerica, MA, USA) with attenuated total reflectance (ATR) mode was used to acquire Fourier transform infrared spectra (ranging from 4000 to 500 cm^−1^). The total number of scans used to record the FTIR spectra was 1024 with 4 cm^−1^ resolution. SEM images were taken using a FEI QuantaTM 650 FEG scanning electron microscope (Thermo Fisher Scientific, Hillsboro, OR, USA) at 20 kV beam voltage. Hardness was measured using an MTS NanoIndenter XP with a Berkovich diamond tip (nominal radius 50 nm). The calibration of the indenter area function before and after hardness measurements was tested for the fused silica standard (accepted Young’s modulus of 72 GPa) to confirm that the tip geometry did not change during the testing of all samples. All indents, including those on silica, were made to a maximum depth of 150 nm. The measured hardness was determined at maximum load. The range of measured silica before/after Young’s modulus values was found to be consistent with the accepted value, and therefore the indenter tip area function was determined not to have changed significantly during actual sample testing.

### 2.3. Density Functional Theory

Density function theory (DFT) [27,28] calculations were performed with the Vienna ab initio simulation package (VASP) version 5.4.4 [29,30], using plane-wave basis sets and the pseudopotential method. The projector augmented wave (PAW) method [31,32] and Perdew–Burke–Ernzerhof generalized gradient approximation (GGA) functional [33] were chosen to conduct the DFT calculations. The wavefunctions were expended with a kinetic energy cutoff of 600 eV. A Γ-centered 10 × 10 × 6 Monkhorst-Pack grid [34] was used in the Brillouin zone sampling. In the structural relaxation, all structures were relaxed until the force on each atom was less than 10^−3^ eV/Å. The convergence criterion of the electronic loop was set to be 10^−6^ eV/unit cell. The phonon calculations were performed using the VASP and PHONOPY [35] packages. The force constants were obtained by density functional perturbation theory implemented in VASP. By Fourier transforming the force constants, PHONOPY can construct the dynamical matrix at an arbitrary q-vector. The phonon dispersion in turn can be calculated by diagonalizing the dynamical matrices. For mechanical properties, the elastic constants were computed directly by VASP. The bulk and shear moduli were then derived by the Voigt–Reuss–Hill averaging method. The theoretical Vickers hardness was then calculated by Chen’s model [36]. The structural visualization and XRD patterns were obtained by VESTA software version 3.4.3 [37].

## 3. Results

### 3.1. X-ray Photoelectron Spectroscopy

**Figure 2:**
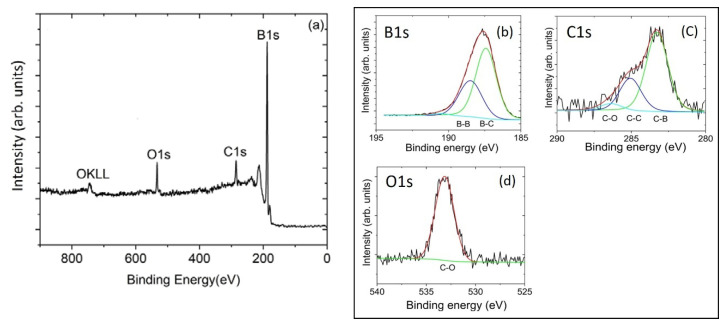
X-ray photoelectron spectroscopy survey scans of (**a**) the BC-1 coating with an elemental composition of 88.1% B, 8.7% C, and 3.2% O. Panels (**b**–**d**) show high-resolution scans for B1s, C1s, and O1s, respectively, with corresponding peaks assigned to B-C, B-B, and C-C (adventitious carbon) bonding. Sample BC-1 also shows bonding associated with C-O.

**Figure 3:**
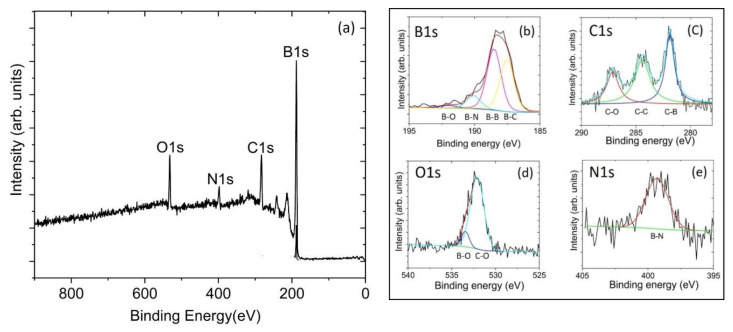
X-ray photoelectron spectroscopy survey scans of (**a**) the BC-2 coating with an elemental composition of 81.0% B, 12.6% C, 4.4% O, and 2.0% N. Panels (**b**–**e**) show high-resolution scans for B1s, C1s, O1s, and N1s, respectively, with corresponding peaks assigned to B-C, B-B, C-C (adventitious carbon), and C-O bonding. Sample BC-2 also shows bonding associated with B-N and B-O.

The XPS of the BC-1 sample showed that the surface is composed of 88.1% B, 8.7% C, and 3.2% O (rel. at%) with no other elements present. A small amount of surface contamination due to adventitious carbon is generally present in samples that have been exposed to air. The complete peak assignments with binding energy is given in Table 1. The high-resolution B1s scan in Figure 2b shows that 75% of the boron is B-C bonded and the remaining 25% is B-B bonded [38,39]. The high-resolution C1s scan in Figure 2c shows that 9% of the carbon is C-O bonded and the high-resolution O1s scan also shows C-O bonding in Figure 2d [40,41]. Figure 2c also shows that 22% of the carbon is C-C bonded and the remaining 69% is B-C bonded [38,39]. Using this information, our XPS measured carbon content in the B-C bonded BC-1 sample is 6%. The XPS of the BC-2 sample shows that the surface is composed of 81.0% B, 12.6% C, 4.4% O, and 2.0% N (rel. at%). The complete peak assignments with binding energy is given in Table 1. The high-resolution B1s scan in Figure 3b shows that 39% of the boron is B-C bonded, 48% of the boron is B-B bonded, 10% of the boron is B-N bonded, and the remaining 3% of the boron is B-O bonded [38,39]. Corresponding peak deconvolution for O1s and N1s reveals B-O and B-N bonding in Figure 3d,e, respectively. [42]. The high-resolution C1s scan in Figure 3c shows that 26% of the carbon is C-O bonded and the high-resolution O1s scan also confirms C-O bonding in Figure 3d [40,41]. According to the high-resolution C1s scan of the BC-2 sample in Figure 3c, 38% of the carbon is C-C bonded and the remaining 36% is B-C bonded [38,39]. Using this information, our XPS measured carbon content in the B-C bonded BC-2 sample is 4.5%. The stoichiometric ratio for B_50_C_2_ should be 96% B, 4% C, and the estimated carbon content in the part of the sample that contains B–C bonding is 4.5%, which is close to the 4% expected.

### 3.2. X-ray Diffraction

**Figure 4:**
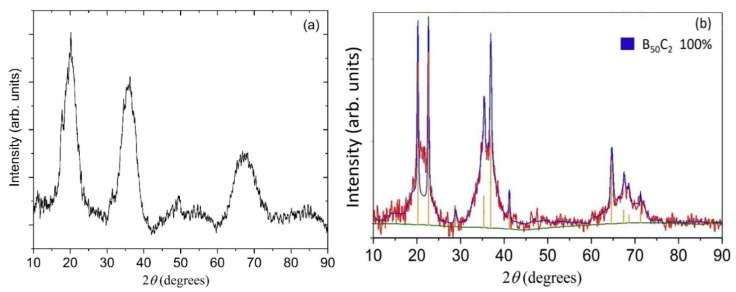
Glancing angle X-ray diffraction patterns for (**a**) sample BC-1 and (**b**) sample BC-2. The X-ray wavelength corresponds to Cu K-α emission (λ = 1.54187 Å). For BC-2, the red curve is the raw data and the blue curve is the Rietveld refinement with lattice parameters a = 8.721 Å, c = 5.058 Å, and α = β = γ = 90°.

The crystal structure of the prepared coatings was evaluated using glancing angle X-ray diffraction (XRD). Figure 4a,b shows XRD patterns for BC-1 and BC-2, respectively. The BC-1 sample shows several broad peaks. The broadened peaks for the BC-1 sample suggest a disordered or nanocrystalline nature for this boron carbide coating. The XRD pattern for the BC-2 sample shows sharp peaks located at the same positions as the centers of the broad peaks of the BC-1 sample. The BC-2 sample also reveals a broad background superimposed onto the sharp peaks. Rietveld refinement was used to analyze the pattern for BC-2, as shown by the blue line in Figure 4b. The experimental pattern was matched to the stoichiometric composition of B_25_C with the chemical structure of B_50_C_2_ with lattice parameters a = 8.721 Å, c = 5.058 Å, as determined using HighScore Plus software, resulting in a weighted profile R-value (Rwp) of 10.248. It should be noted that existing XRD databases are not exhaustive for the wide range of boron-rich carbide structures (including those of B_50_C_2_) and several novel structures are still being reported experimentally or via computational approaches [9,20,44]. For this reason, the lattice constants determined from the performed Rietveld refinement are provided only for comparison with potential structures (e.g., tetragonal vs. orthorhombic B_50_C_2_) modeled by DFT calculations. 

### 3.3. Raman Spectroscopy 

**Figure 5:**
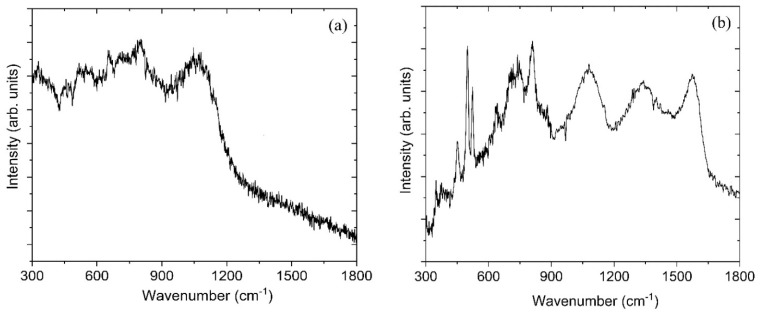
Raman spectra of (**a**) BC-1 and (**b**) BC-2 samples acquired in the range 300–1800 cm^−1^.

Figure 5a shows the Raman spectra of sample BC-1. It is known that Raman-active lattice vibrations are affected much more by structural distortion than those of IR-active vibrations [45]. The spectrum of the BC-1 sample shows a combination of broad bands. The broad bands are typically more characteristic of an amorphous or a partially disordered structure. Figure 5b shows the Raman spectra of the BC-2 sample, revealing two broad bands c.a. 1340 cm^−1^ and 1577 cm^−1^. These bands are commonly associated with disordered carbon and are often referred to as the “D” and “G” bands, respectively. These two bands indicate disordered sp^2^- and sp^3^-bonded carbon in the BC-2 sample and they have been reported in the literature for the deposition of boron carbide material [7,16,21,46,47]. The Raman spectrum of the BC-2 sample matches well with that found by Gao et al. in 2019 for B_50_C_2_ [7].

### 3.4. Fourier Transform Infrared Spectroscopy (FTIR)

**Figure 6:**
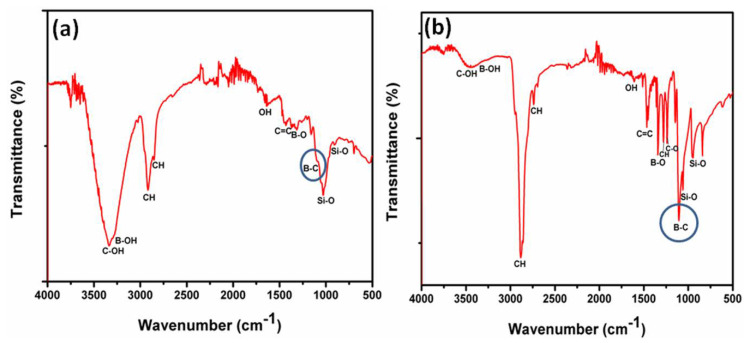
Fourier transformed infrared spectroscopy spectral analysis of the (**a**) BC-1 and (**b**) BC-2 samples.

To enable more insight into the nature of the chemical bonding present in the boron carbide coatings, FTIR spectral analysis was carried out, as shown in Figure 6. The complete peak assignment with transmittance frequency is also given in Table 2 and Table 3 for BC-1 and BC-2 respectively. 

The FTIR spectra of BC-1 and BC-2 exhibit several peaks in the fingerprint region (1500–500 cm^−1^). The major IR peaks of both BC-1 and BC-2 were assigned appropriately after cross-referencing with previously reported infrared studies of boron carbon chemical bonds [18,48,49]. Interestingly, BC-2 reveals more pronounced and sharper bands in comparison with BC-1. This can be attributed to the crystalline nature of the BC-2 coating (which may endow a more ordered arrangement of boron and carbon atoms) in comparison with the disordered nature of BC-1. The major peak of interest for both BC-1 and BC-2 is around 1100 cm^−1^ (more specifically 1097 and 1107 cm^−1^ for BC-1 and BC-2, respectively). The peak near 1100 cm^−1^ has been assigned to intra-icosahedral vibrations of the B-C bond [50]. This major peak is sharper and more pronounced for BC-2 compared to BC-1. In addition to the boron–carbon bonding, there is the presence of other types of chemical bonds for both BC-1 and BC-2, such as C-O, C=C, C-H, B-O, and OH. The obtained peaks were found to be consistent with respect to the infrared spectrum of the boron–carbon bonds by Romanos et al. [49]. The peaks around 1022 cm^−1^, 1058 cm^−1^, 992 cm^−1^ (Si-O), and 700 cm^−1^ (Si-C) can be attributed to the contribution of the silicon substrate used to deposit the BC coating [51]. 

### 3.5. Scanning Electron Microscopy (SEM)

**Figure 7:**
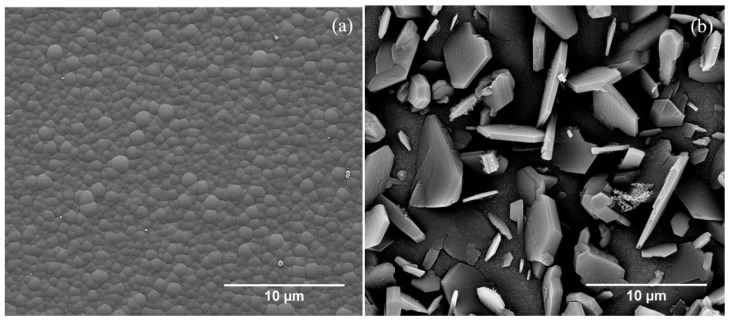
Scanning electron microscopy (SEM) images of samples (**a**) BC-1 and (**b**) BC-2. The much higher crystallinity of BC-2 is apparent from large facets with very fine-grained morphology between facets.

Scanning electron microscopy (SEM) was used to image the surface morphology of the BC-1 and BC-2 coating surfaces, as shown in Figure 7a,b, respectively. The surface morphology of these coatings is quite different. The BC-1 surface shows densely packed rounded nodules without any strong indication of crystalline facets. In contrast, the BC-2 coating shows large faceted platelets protruding from a very fine-grained base structure. There does not appear to be any preferred orientation of the crystalline facets and a few crystals appear to be twinned. 

### 3.6. Nanoindentation

**Figure 8:**
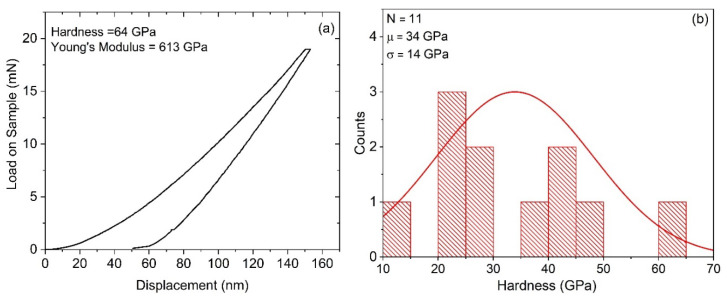
(**a**) Nanoindentation load–displacement curve from one location on the BC-1 coating at a depth of 150 nm. The extracted values of nanoindentation hardness and Young’s modulus are also indicated. (**b**) Histogram from 11 indents showing hardness values for sample BC-1.

Figure 8a shows the nanoindentation load/displacement data of sample BC-1. The indents were performed to a depth of 150 nm at several locations (N = 11 indents) on the film. The average hardness of all indents was 34 GPa with several measurements greater than 40 GPa. The indent with the highest measured hardness was 64 GPa and it had Young’s modulus of 614 GPa. The average Young’s modulus of this sample was 411 GPa. The relative contribution of elastic and plastic deformation can be calculated from the final unloading depth of the load–displacement curves. A high elastic recovery (about 67%) from the boron carbide films was measured from the unloading data. Figure 8b shows the large spread in the hardness data for sample BC-1. Factors which are likely to influence this spread include sample surface roughness and non-homogeneity in material properties at the scale of nanoindentation. Due to the much higher surface roughness of the crystalline sample (BC-2) and the corresponding difficulty in obtaining valid indented surface finds, this sample was not tested.

### 3.7. Density Functional Theory Calculations

To better understand the crystalline sample BC-2, we first performed DFT calculations using B_50_C_2_ structures reported by Ploog et al. [52] and Will et al. [53] without structural relaxation, where two interstitial boron atoms are located at the Wyckoff 8h or 8i sites, and two carbon atoms at the 2b sites. Our phonon dispersion calculations for these structures have negative frequency modes (not shown). Even if the boron atoms are allowed to relax away from the high-symmetry Wyckoff 8h or 8i positions, the negative modes still exist. These results indicate dynamical instability of these previously reported structures.

**Figure 9:**
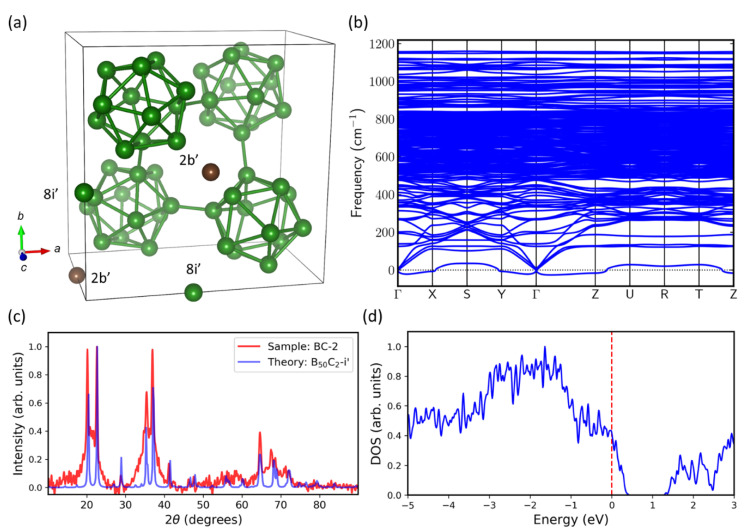
(**a**) The second lowest energy structure found in our density functional theory (DFT) calculations with full structure relaxation, B50C2-8i’. The tetragonal symmetry remains intact after relaxation. The positions of two carbon atoms move from 2b (0, 0, 0.5) to 2b’ (0, 0, 0.4855), and two boron atoms move slightly from 8i (0.4334, 0, 0) to 8i’ (0.4395, −0.0019, −0.0091). (**b**) Phonon dispersion of B_50_C_2_-8i’ shows its dynamical instability. (**c**) Theoretical B_50_C_2_-8i’ X-ray diffraction (XRD) pattern (blue) compared with experimental XRD pattern of the sample BC-2 (red). (**d**) Electronic calculations show finite DOS of B_50_C_2_-8i’ at the Fermi level (red dashed line).

**Figure 10:**
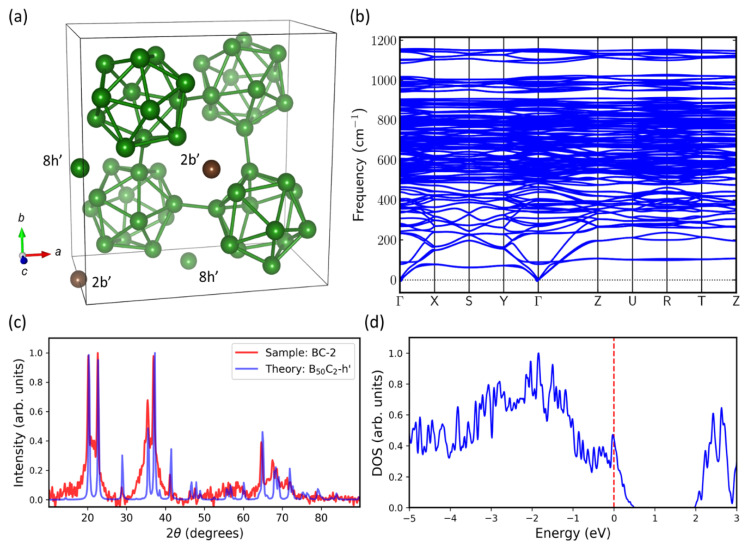
(**a**) The lowest energy structure found in our DFT calculations, B_50_C_2_-8h’. After full structure relaxation, the crystal symmetry changes from tetragonal to orthorhombic. The positions of the two carbon atoms move from 2b (0, 0, 0.5) to 2b’ (0, 0, 0.503), and the two boron atoms move from 8h (0, 0.5, 0.8557) to 8h’ (0, 0.5, 0.7908). (**b**) Phonon dispersion of relaxed B_50_C_2_-8h’ shows that it is dynamically stable. (**c**) Theoretical B_50_C_2_-8h’ XRD pattern (blue) compared to the experimental XRD pattern of sample BC-2 (red). (**d**) Electronic calculations show finite DOS of B50C2-8h’ at the Fermi level (red dashed line).

We next considered Will’s B_50_C_2_-8i (two borons at 8i) and B_50_C_2_-8h (two borons at 8h) as initial structures, and then performed full structural relaxation calculations that allowed both unit cell and atomic positions to vary. Figure 9a shows the structure of B_50_C_2_-8i’ (obtained by fully relaxing B_50_C_2_-8i), which has the second lowest energy in our study (with a formation energy = +3 meV/atom compared to the B_50_C_2_-8h’ structure shown in Figure 10a). In B_50_C_2_-8i’, the two interstitial boron atoms relax from 8i (0.4334, 0, 0) to 8i’ (0.4395, −0.0019, −0.0091), and two carbon atoms from 2b (0, 0, 0.5) to 2b’ (0, 0, 0.4855). Based on the phonon spectra in Figure 9b, B_50_C_2_-8i’ is still dynamically unstable. In Figure 9c, we compare the theoretical B_50_C_2_-8i’ and experimental BC-2 sample XRD patterns. The main theoretical XRD peaks are in good agreement with the experiment. Figure 9d shows the electronic density of states (DOS) for B_50_C_2_-8i’, which is metallic with a finite DOS at the Fermi level. In our calculations, B_50_C_2_-8i’ has bulk and shear moduli equal to 212 and 169 GPa, respectively. By means of Chen’s empirical model [36], the Vickers hardness is found to be 28 GPa. We note that the relaxed B_50_C_2_-8i’ structure remains tetragonal, which suggests that a dynamically stable structure might exist with a lower symmetry. 

Figure 10a shows the structure of B_50_C_2_-8h’ (obtained by fully relaxing B_50_C_2_-8h), which becomes orthorhombic with lattice parameters a = 8.6772 Å, b = 8.7237 Å, and c = 5.0517 Å. This structure has the lowest energy in our study (with a formation energy = 0 meV/atom). In B_50_C_2_-8h’, the two interstitial boron atoms relax from 8h (0, 0.5, 0.8557) to 8h’ (0, 0.5, 0.79588), and the two carbon atoms from 2b (0, 0, 0.5) to 2b’ (0, 0, 0.5033). Since no negative mode exists in the phonon dispersion in Figure 9b, B_50_C_2_-8h’ is dynamically stable. Figure 10c compares the XRD patterns of the theoretical B_50_C_2_-8h’ structure and the BC-2 sample, which also shows a good theory–experiment agreement. The DOS calculations for B_50_C_2_-8h’ in Figure 10d indicate its metallicity. The theoretical bulk and shear moduli, as well as Vickers hardness for B_50_C_2_-8h’, were computed to be 213 GPa, 172 GPa, and 29 GPa, respectively. These values are similar to those for the B_50_C_2_-8i’ structure in Figure 9a.

## 4. Discussion

The boron carbides exist as a single-phase material from about 8 at.% carbon to about 20 at.% carbon [4,5]. Very few studies have reported on the atomic structures of boron carbide in the regime below ~13.3 at.% C (known as boron–very rich boron carbide, BvrBC) due to the complexity of the structure and bonding [9,54]. A model based on XRD data proposes that the 20 at.% carbon composition is made up of B_12_ icosahedra and carbon–carbon–carbon (CCC) chains [55]. Other models also show that, at 20 at.% carbon composition, a carbon atom is inserted into the icosahedra as B_11_C and can also make carbon–boron–carbon (CBC) chains [56,57]. As the atomic percentage of carbon decreases below 20% to around 13.3%, boron preferentially replaces carbon in the icosahedron to form B_12_. Carbon atoms only remain in the icosahedral chain when their atomic percentage is around 13.3% [6,58,59,60]. The atomic percentage of carbon is below 13.3% in our samples; and B-C bonded carbon is only 6.1% and 4.5% in BC-1 and BC-2, respectively. The atomic similarity between boron and carbon makes it difficult to distinguish their atomic locations in most characterization techniques. For boron carbides, Raman bands in the range of 600–1200 cm^−1^ correspond to vibrations involving icosahedral atoms. Two sharp peaks at 481 cm^−1^ and 534 cm^−1^ are assigned to vibrations related to the CBC chain and a broad band centered at 400 cm^−1^ represents the CBB chain [4]. For the BC-1 sample, we observe two broad bands in the 600–1200 cm^−1^ range and no peaks that would correspond to the C-B-C or C-B-B chain formation. Instead, this sample shows broad peaks indicative of a disordered structure. Broadened peaks in the Raman spectra throughout the entire range investigated indicates disorder in the B_12_ icosahedra. Such broadening can be due to structural defects, such as micro-twinning [22]. 

The BC-2 sample does show the two sharp Raman peaks around 481 cm^−1^ and 534 cm^−1^ associated with vibrations related to the CBC chain, along with broadened peaks throughout the entire wavenumber range. It is notable that the SEM of this sample shows microstructural twinning on crystalline facets as well as very fine-grained material between facets. This may explain the broad base to the peaks in the XRD data (Figure 4b). Different stoichiometric boron carbide structures, such as B_4_C and B_50_C_2_, result in distinctly different locations of boron atoms in the unit cell. Boron atoms in B_4_C belong to an icosahedron with a carbon chain in B_4_C or one boron atom in the icosahedron can be substituted by a carbon atom and form a C-B-C chain. In contrast, some of the boron atoms in B_50_C_2_ are in single atom form and take positions in interstitial sites, with carbon being isolated [7].

In the literature, two separate studies from Ploog et al. [52] and Will et al. [53] have reported two tetragonal structures with different lattice parameters. Ploog’s structure has lattice parameters a = b = 8.722 Å and c = 5.080 Å, while Will’s structure has a = b = 8.753 Å and c = 5.093 Å. In our DFT calculations, the phonon spectra of both structures show negative phonon modes, indicating their dynamical instabilities. In our calculations with fixed lattice parameters but relaxed Wyckoff positions, the boron atoms at 8h (0, 0.5, z) change dramatically from z = 0.8557 to z = 0.7580, while those at 8i (x, 0, 0) move only slightly from x = 0.4334 to 0.4363. However, these structures are still dynamically unstable even with relaxed Wyckoff positions.

In our subsequent DFT calculations that fully relax both lattice parameters and Wyckoff positions, the structure B_50_C_2_-8i’ (Figure 9a) remains in the tetragonal symmetry, with the lattice parameters change to a = b = 8.6906 Å and c = 5.0827 Å. The Wyckoff positions of two boron atoms move to 8i’ (0.4395, −0.0019, −0.0091) and two carbon atoms to 2b’ (0, 0, 0.4855). However, this fully relaxed tetragonal structure B_50_C_2_-8i’ still shows negative phonon modes. In short, a tetragonal B_50_C_2_ appears dynamically unstable in DFT calculations.

On the other hand, for B_50_C_2_-8h’ (Figure 10a), the crystal symmetry changes to orthorhombic (a = 8.6772 Å, b = 8.7237 Å, c = 5.0517 Å), with the two boron atoms located at 8h’ (0, 0.5, 0.79588) and two carbons at 2b’ (0, 0, 0.5033). Importantly, this orthorhombic structure is dynamically stable. This B_50_C_2_-8h’ is also the most stable structure (formation energy = 0 meV/atom) in DFT. Experimentally, an effective tetragonal phase might be observed due to a statistical average, as the two interstitial boron atoms could be randomly placed onto any two of the 8h sites.

In our MPCVD system, we find that higher atomic boron and BH emissions were measured by optical emission spectroscopy (OES) at lower pressure and higher power. We used this fact in order to grow the boron-rich coatings in this study. In addition, subtle changes in the sample location within the plasma (determined by the molybdenum screw design, as shown in the inset of the Figure 1), which has a substantial effect on the shape of the plasma near the sample surface and on the corresponding coating structure. The control of the coatings’ degree of crystallinity was achieved by manipulating the sample stage design (and thus the proximity of the sample to the plasma). Presumably, this affects the active growth species nearest the sample and allows for the control of the film structure. Future work will involve an investigation of spatially resolved optical emission spectroscopy to identify the spatial variation of excited state species near the growth surface. It is therefore very important to acknowledge and further investigate the strong influence that even very minor adjustments in sample proximity to the plasma have on the resulting film structure by MPCVD. Given that all other controlled deposition parameters were held fixed in this study (including substrate temperature to within 25 °C), we believe this allows a new parameter space to be carefully explored. In the current study, we have synthesized two boron-rich carbide structures yielding very different growth morphology and structural forms, with all other deposition parameters being equal. We plan to further take advantage of this parameter space that involves sample proximity to the plasma to create other unique superhard boron structures, such as BC_2_N, B_2_C_3_N, B_4_C_5_N_2_, and B_5_C_3_N.

In the studies of boron-rich materials, there exists a known discrepancy between experiment and theory, which was dubbed the metal/insulator problem by Uemura et al. [61]. Basically, band theory predicts several boron-rich materials like β-boron and B_13_C_2_ to be metallic. In experiments, however, these materials are found to be semiconductors [62]. This problem is related to the lack of ordering in interstitial atoms residing between B_12_ icosahedra [61]. Such structures have been regarded as geometrically frustrated systems. In DFT, as long as the two carbon atoms are placed at the 2b sites, B_50_C_2_ is metallic. One possible way to open an energy gap at the Fermi level is to insert one carbon into one of the four B_12_ icosahedra [9]. Such metastable structures have formation energies around 15 meV/atom above the ground state, which is still well below the thermal energy scale during synthesis (825 °C~100 meV/atom). Therefore, from the viewpoint of thermodynamics, several different stable and metastable B_50_C_2_ phases could possibly exist to various extents during the chemical vapor deposition. However, resolving the metal/insulator problem is beyond the scope of our current research.

## 5. Conclusions

Superhard boron-rich carbide coatings were deposited in a microwave plasma chemical vapor deposition (MPCVD) system. The control of the coatings’ degree of crystallinity was achieved by manipulating the sample stage design (and thus the proximity of the sample to the plasma). Amorphous boron-rich carbide coatings were grown on a flat sample holder, whereas crystalline boron-rich carbide coatings were formed on a sample holder with a shallow inset. XRD, Raman spectroscopy, and SEM were used to identify and characterize the amorphous or crystalline nature of boron-rich carbide coatings. Rietveld refinement of the crystalline sample led to the prediction of a B_50_C_2_-type structure. Hardness values of the amorphous sample are as high as 64 GPa. Nanoindentation measurements yield hardness values that vary considerably from point to point on the surface, so future work can focus on improving the homogeneity of the coatings. In our DFT calculations, tetragonal B_50_C_2_ structures were found to be dynamically unstable. The only dynamically stable structure, which is also the lowest energy structure, has an orthorhombic symmetry. In theory, unless carbon atoms are inserted directly into the B_12_ icosahedron, B_50_C_2_ crystalline phases are metallic. The ability to better control and understand the morphology, structure, and electronic property of B_50_C_2_ by microwave plasma chemical vapor deposition will enable further applications with superhard boron-rich boron carbide materials.

## Figures and Tables

**Table 1:** X-ray photoelectron spectroscopy compositional analysis and fitted parameters of B1s, C1s, O1s, and N1s of BC-1 and BC-2 samples. “Data from reference [38,39,40,41,42,43].”

Sample	Peaks	Binding Energy	Peak Area (%)	Assignment
**BC-1**	B1s	186.2	75	B-C
	B1s	187.4	25	B-B
	C1s	283.3	69	C-B
	C1s	284.9	22	C-C
	C1s	286.5	9	C-O
	O1s	533.3	100	C-O
**BC-2**	B1s	187.0	39	B-C
	B1s	188.5	48	B-B
	B1s	190.5	10	B-N
	B1s	192.0	3	B-O
	C1s	282.0	26	C-B
	C1s	284.5	36	C-C
	C1s	287.0	38	C-O
	O1s	532.3	90	C-O
	O1s	533.6	10	B-O
	N1s	399.0	100	B-N

**Table 2:** Complete peak assignment for the Fourier transformed infrared spectroscopy (FTIR) spectral analysis of the BC-1 coating.

Transmittance Frequency (cm^−1^)	Assignment
1022	Si-O stretching vibrations (from the silicon substrate)
1097	B-C stretching vibrations
1305	B-O stretching vibrations
1450	C=C stretching vibrations
1622	OH bending modes of C-OH and B-OH groups
2857	CH symmetric stretching vibrations
2910	CH asymmetric stretching vibrations
3272	B-OH stretching vibrations
3440	C-OH stretching vibrations

**Table 3:** Complete peak assignment for the FTIR spectral analysis of the BC-2 coating.

Transmittance Frequency (cm^−1^)	Assignment
1058	Si-O stretching vibrations (from the silicon substrate)
1107	B-C stretching vibrations
1236	C-O stretching vibrations
1292	C-H bending vibrations
1340	B-O stretching vibrations
1450	C=C stretching vibrations
1616	OH bending modes of C-OH and B-OH groups
2750	CH symmetric stretching vibrations
2879	CH asymmetric stretching vibrations
3274	B-OH stretching vibrations
3442	C-OH stretching vibrations
